# Effectiveness of standardized nursing care plans to achieve A1C, blood pressure, and LDL-C goals among people with poorly controlled type 2 diabetes mellitus at baseline: four-year follow-up study

**DOI:** 10.1186/s12875-018-0800-z

**Published:** 2018-07-24

**Authors:** J. Cárdenas-Valladolid, A. López-de Andrés, R. Jiménez-García, M. J. de Dios-Duarte, P. Gómez-Campelo, C. de Burgos-Lunar, F. J. San Andrés-Rebollo, J. C. Abánades-Herranz, M. A. Salinero-Fort

**Affiliations:** 1 Dirección Técnica de Sistemas de Información, Gerencia Asistencial de Atención Primaria, Servicio Madrileño de Salud, C/ San Martín de Porres, 6, 28035 Madrid, Spain; 2 Universidad Alfonso X el Sabio, Villanueva de la Cañada, Madrid, Spain; 3 Aging and Fragility in the Elderly Group, Hospital La Paz Institute for Health Research (IdiPAZ), Madrid, Spain; 4 MADIABETES Research Group, Madrid, Spain; 5 Facultad de Ciencias de la Salud, Universidad Rey Juan Carlos, Alcorcón, Madrid, Spain; 6 Jefatura de Estudios del Grado en Enfermería, Universidad Alfonso X el Sabio, Villanueva de la Cañada, Madrid, Spain; 7 Innate Immunity Group, Hospital La Paz Institute for Health Research (IdiPAZ), La Paz University Hospital, Madrid, Spain; 8 University Centre of Health Sciences San Rafael-Nebrija, Antonio de Nebrija University, Madrid, Spain; 9 Dirección General de Salud Pública, Subdirección de Promoción, Prevención y Educación de la Salud, Consejería de Sanidad, Madrid, Spain; 10 Red de Investigación en Servicios de Salud en Enfermedades Crónicas (REDISSEC), Madrid, Spain; 11 Centro de Salud Las Calesas, Madrid, Spain; 12 Centro de Salud Monóvar, Madrid, Spain; 13 Subdirección General de Investigación. Consejería de Sanidad, Madrid, Spain

**Keywords:** Patient care planning, Diabetes mellitus type 2, Prospective studies, Outcome assessment, NANDA, NIC

## Abstract

**Background:**

No studies that have measured the role of nursing care plans in patients with poorly controlled type 2 diabetes mellitus. Our objectives were firstly, to evaluate the effectiveness of implementing Standardized languages in Nursing Care Plans (SNCP) for improving A1C, blood pressure and low density lipoprotein cholesterol (ABC goals) in patients with poorly controlled type 2 diabetes mellitus at baseline (A1C ≥7%, blood pressure ≥ 130/80 mmHg, and low-density lipoprotein cholesterol≥100 mg/dl) compared with Usual Nursing Care (UNC). Secondly, to evaluate the factors associated with these goals.

**Methods:**

A four-year prospective follow-up study among outpatients with type 2 diabetes mellitus**:** We analyzed outpatients of 31 primary health centers (Madrid, Spain), with at least two A1C values (at baseline and at the end of the study) who did not meet their ABC goals at baseline. A total of 1916 had A1C ≥7% (881 UNC versus 1035 SNCP). Two thousand four hundred seventy-one had systolic blood pressure ≥ 130 mmHg (1204 UNC versus 1267 SNCP). One thousand one hundred seventy had diastolic blood pressure ≥ 80 mmHg (618 UNC versus 552 SNCP); and 2473 had low-density lipoprotein cholesterol ≥100 mg/dl (1257 UNC versus 1216 SNCP). Data were collected from computerized clinical records; SNCP were identified using NANDA and NIC taxonomies.

**Results:**

More patients cared for using SNCP achieved in blood pressure goals compared with patients who received UNC (systolic blood pressure: 29.4% versus 28.7%, *p* = 0.699; diastolic blood pressure: 58.3% versus 53.2%, *p* = 0.08), but the differences did not reach statistical significance. For A1C and low-density lipoprotein cholesterol goals, there were no significant differences between the groups. Coronary artery disease was a significant predictor of blood pressure and low-density lipoprotein cholesterol goals.

**Conclusions:**

In patients with poorly controlled type 2 diabetes mellitus, there is not enough evidence to support the use of SNCP instead of with UNC with the aim of helping patients to achieve their ABC goals. However, the use of SNCP is associated with a clear trend of a achievement of diastolic blood pressure goals.

## Background

In Spain, approximately 6 million people have diabetes mellitus (DM) [1], and this number is increasing annually [2]. DM represents a major public health problem because it is a well-known risk factor for stroke [3], coronary artery disease (CAD) [4] and cardiovascular disease [5].

Poor control of blood pressure (BP), lipids, and glycosylated hemoglobin (A1C) is strongly associated with adverse outcomes in patients with type 2 DM (T2DM) [6]. The American Diabetes Association (ADA) recommends that patients with DM achieve their ABC goals, namely, A1C < 7%, BP < 130/80 mmHg, and low density lipoprotein cholesterol (LDL-C < 100 mg/dl [7]. However, at least one-third of patients with T2DM [8] fail to achieve their ABC goals.

The responsibility for the care of patients with T2DM in Spain has shifted to multidisciplinary teams based in primary health care (PHC) settings that are composed mainly of family doctors and nurses.

Achieving ABC goals for T2DM patients depends on several factors, such as physical activity levels [9], stress reduction [10], medication adherence [11], and meal plans [12]. These targets form the basis of wide range of interventions implemented by nurses and aimed at improving diabetes care and achieving metabolic control [13].

In the last decade, there has been a considerable improvement in Standardized languages in Nursing Care Plans (SNCP) with NANDA-International [14] Nursing Diagnoses and Interventions (NIC) [15]. Since 1998, these taxonomies have been progressively incorporated into clinical practice and computerized clinical records (CCR) in Madrid (Spain). However they are still not used by 100% of nursing staff [16].

Our group recently established the effectiveness of SNCP for improving health outcomes for T2DM patients [17]. However, to our knowledge, no studies have measured the role of nursing care plans in patients with poorly controlled T2DM. Accordingly, we hypothesize that SNCP may be effective in helping patients with poorly controlled T2DM to achieve their ABC goals. We also consider that it is necessary to know the magnitude of the effect of SNCP and to compare it with that of other therapeutic strategies.

Our study had two objectives. First, we evaluated the effectiveness of SNCP as a component of CCR registration for helping patients with poorly controlled T2DM at baseline (A1C ≥7%, blood pressure ≥ 130/80 mmHg, and LDL-C ≥ 100 mg/dl) to achieve their ABC goals and compared our findings with those of Usual Nursing Care (UNC), provided by dedicated trained nurses in PHC settings, second, we evaluated the factors associated with meeting ABC goals.

## Methods

This study was conducted as part of a broader research project which is described in detail elsewhere [17, 18]. A prospective cohort follow-up study was carried out between March 2008 and February 2012 in T2DM patients attending follow-up appointments with a nurse at PHC centers.

The two types of nursing care plans implemented: SNCP (*n* = 2105) and UNC (n = 2105) were delivered by registered nurses trained in diagnostic reasoning based on NANDA-I and NIC taxonomies and working in 31 PHC centers in the northeastern area of the city of Madrid, Spain.

Eligibility criteria for patients were: age ≥ 30 years with at least two records in the CCR during the previous year and an International Classification of Primary Care [19] code indicating T2DM (T90). Patients were not selected if they met any of the following exclusion criteria: gestational diabetes, being homebound, and a life expectancy of less than 1 year (according to the physician’s clinical judgment).

The number of patients selected for this study was lower than in the our previous study [17], as we preferred to restrict our analysis to patients from the SNCP group with at least two A1C values (baseline and end of study) over the four-year follow-up (*n* = 2105) Therefore, we decided to select a random sample of an equal size in the UNC group (*n* = 2105).

**Fig. 1:**
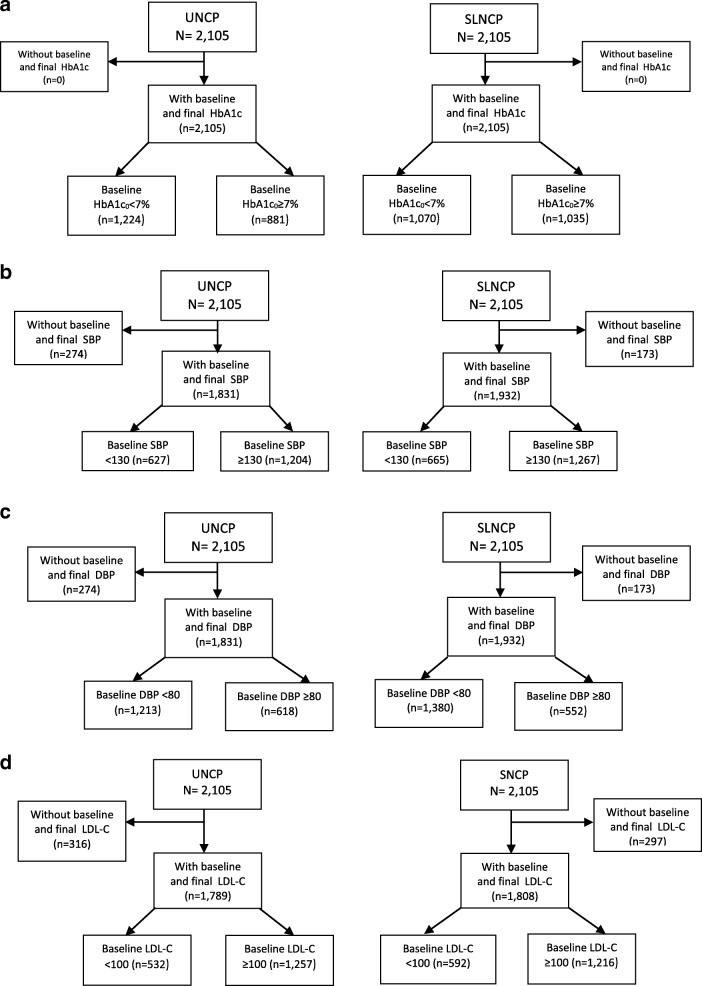
Procedure and patients included in the study, stratified by ABC goal. ABC goals: (**a**) HbA1c < 7%, (**b**) SBP < 130 mmHg, (**c**) DBP < 80 mmHg and (**d**) LDL-C < 100 mg/dl; UNCP: Usual Nursing Care; SNCP: Standardized languages in Nursing Care Plans; HbA1c: glycosylated hemoglobin; DBP: diastolic blood pressure; SBP: systolic blood pressure; LDL-C: low-density lipoprotein-cholesterol

Figure 1 provide details of the study procedure, patients recruitment and exclusion, and patients without baseline and final A1C, LDL cholesterol and BP values Only those with poor diabetes control were finally included.

The sample size was calculated taking into account a UNC: SNCP ratio of 1:1.10 (estimated a proportion of 30% patients with A1C < 7% in the UNC group, alpha risk of 0.05 and a beta risk of 0.20 in a two-sided test). A sample size of 873 UNC patients and 1029 SNCP patients is necessary to recognise a statistically significant relative risk of good glycemic control (A1C < 7%) of ≥1.22 in the UNC vs. the SNCP group. A drop-out rate of 0.05 was anticipated.

We only analyzed patients who did not meet their ABC goals at baseline: A1C ≥7% (*n* = 1916; 881 with UNC and 1035 with SNCP), systolic blood pressure (SBP) ≥130 mmHg (*n* = 2471; 1204 with UNC and 1267 with SNCP), diastolic blood pressure (DBP) ≥80 mmHg (*n* = 1170; 618 with UNC and 552 with SNCP) and LDL-C ≥ 100 mg/dl (*n* = 2473; 1257 with UNC and 1216 with SNCP).

### Measures

Data were collected under routine clinical practice conditions from CCR at PHC centers in the Madrid Health Service and processed using OMI-AP software., The CCR was previously validated for patients with a diagnosis of T2DM [20].

SNCP was identified based on the following three criteria:

- Criterion 1. The patient has a CCR code that corresponds to Gordon’s functional health patterns [21] in at least one of the following areas: activity and exercise; nutritional and metabolic; and health perception and health management.
- Criterion 2. The problems identified were described using nursing diagnosis statement codes based on the NANDA-I taxonomy, which is used in T2DM patients. A nursing diagnosis based on NANDA-I taxonomy is defined as a clinical judgment concerning a human response to a health condition/life process, or a vulnerability in that response, by an individual, family, group, or community and provides the basis for selection of nursing interventions to achieve outcomes for which the nurse has accountability [14].
- Criterion 3. The nursing intervention carried out was registered according to the NIC taxonomy codes, which used in T2DM patients [15].

The nurses who applied SNCP used the following domains: nutrition, coping/stress tolerance, life principles, health promotion, self-perception, perception/cognition, activity/rest and safety/protection. The main nursing diagnoses were Imbalanced nutrition: more than body requirements (00001), Non-compliance (00079), Ineffective self-health management (00078), Ineffective family therapeutic régimen management (00080), Health-seeking behaviors: Management DM (00084), Deficient knowledge (00126), Sedentary lifestyle (00168) and Impaired skin integrity (00046). The same nurses also delivered UNC to the control group.

UNC was defined as the treatment and monitoring of T2DM including control of blood sugar, control of cardiovascular risk factors, adherence to drug therapy, health education, change in lifestyle and self-management according to local guidelines [22].

The following variables were recorded: sociodemographic characteristics (gender, age), clinical variables (diabetes over time), personal health habits sucha as smoking (cigarettes/day) and drinking (alcohol units/week), physical activity (measured in hours per week with any exercise or activity outside of the patients’ regular job being considered, and recoded as vigorous-intensity, moderate-intensity, sedentary), associated morbidity (dyslipidemia, hypertension, coronary heart disease), complications of diabetes mellitus (retinopathy, nephropathy, neuropathy), and the type of treatment prescribed (dietary and pharmacological). Biochemical–biological parameters were also collected, as follows: body mass index (BMI), SBP, DBP, total cholesterol, LDL-C, high-density lipoprotein cholesterol (HDL-C), triglycerides, and A1C.

Blood pressure was measured according to the recommendations of the Seventh Report of the Joint National Committee on Prevention, Detection, Evaluation, and Treatment of High Blood Pressure [23]; these recommendations were current at the start of this study.

Cholesterol and triglycerides were determined using enzyme assays. LDL-C was calculated according to the Friedewald formula [LDL-C = total cholesterol (HDL-C + trigycerides/5)] in participants with triglycerides below 400 mg/dL. HDL-C was measured after precipitation of apoB lipoproteins. A1C was measured using a high-performance liquid chromatography.

### Statistical analysis

A descriptive analysis was carried out for each variable included in this study; quantitative variables were expressed as the mean and standard deviation and qualitative variables as. The chi-square test was used to compare the percentage of patients who achieved their ABC goals. The t-test was used for quantitative data. Multiple logistic regression analysis was used to identify the variables associated with each goal (A1C < 7%, DBP < 80 mmHg, SBP < 130 mmHg, and LDL cholesterol < 100 mg/dl) and the relationship between each ABC goal and selected predictor variables was examined. In addition, each logistic regression model was adjusted for all potential confounders, including the variables for which differences between the groups were observed at baseline.

In all instances, the accepted level of significance was 0.05 or less. The 95% confidence interval was reported. All analyses were carried out on an intention-to-treat (ITT) principle. With the analyses were performed using SPSS (SPSS for Windows, V.19.0; IBM Corp, Armonk, New York, USA).

## Results

The demographic and clinical characteristics of patients with poorly controlled T2DM at baseline, stratified by SNCP and UNC groups are shown in Table 1. Among patients with A1C ≥7% at baseline, those in the SNCP group were older, had lived with DM for longer, and had received less treatment with diuretics than patients from the UNC group.

**Table 1:** Demographic and clinical characteristics of patients with poorly controlled T2DM at baseline, stratified by SNCP and UNC

	A1C ≥7% (*n* = 1916)			DBP ≥80 mmHg (*n* = 1170)			SBP ≥ 130 mmHg (*n* = 2471)			LDL Chol ≥100 mg/dl (*n* = 2473)		
	SNCP (*n* = 1035)	UNC (*n* = 881)	*p* value	SNCP (*n* = 552)	UNC (*n* = 618)	*p* value	SNCP (*n* = 1267)	UNC (*n* = 1204)	*p* value	SNCP (*n* = 1216)	UNC (*n* = 1257)	*p* value
Gender female n (%)	581 (56.2)	473 (53.7)	0.27	299 (54.2)	337 (54.5)	0.90	741 (58.5)	677 (56.2)	0.25	684 (56.3)	711 (56.6)	0.89
Age mean (sd)	70 (10)	67.7 (11)	0.00	67.5 (10)	66.3 (10)	0.05	71.5 (8.7)	70.2 (9.3)	0.00	70 (10)	67.9 (10.2)	0.00
Tobacco n (%)	205 (19.8)	190 (21.6)	0.34	120 (21.7)	122 (19.7)	0.40	221 (17.4)	208 (17.3)	0.91	218 (17.9)	238 (18.9)	0.52
CAD n (%)	159 (15.4)	115 (13.1)	0.15	39 (7.1)	49 (7.9)	0.58	151 (11.9)	123 (10.2)	0.18	103 (8.5)	102 (8.1)	0.75
Dyslpidemia n (%)	571 (55.2)	451 (51.2)	0.08	293 (53.1)	323 (52.3)	0.78	684 (54)	616 (51.2)	0.16	682 (56.1)	658 (52.3)	0.06
Retinopathy n (%)	60 (5.8)	53 (6)	0.84	20 (3.6)	16 (2.6)	0.31	60 (4.7)	43 (3.6)	0.15	57 (4.7)	38 (3)	0.03
Nephropathy n (%)	58 (5.6)	47 (5.3)	0.80	28 (5.1)	31 (5)	0.97	78 (6.2)	68 (5.6)	0.59	57 (4.7)	65 (5.2)	0.58
Neuropathy n (%)	21 (2)	24 (2.7)	0.32	7 (1.3)	8 (1.3)	0.97	19 (1.5)	21 (1.7)	0.63	19 (1.6)	19 (1.5)	0.92
Hypertension n (%)	705 (68.1)	624 (70.8)	0.20	429 (77.7)	510 (82.5)	0.04	997 (78.7)	975 (81)	0.16	838 (68.9)	871 (69.3)	0.84
OAD n (%)	885 (85.5)	743 (84.3)	0.48	430 (77.9)	452 (73.1)	0.06	1008 (80)	902 (74.9)	0.00	924 (76)	884 (70.3)	0.00
Insulin n (%)	344 (33.2)	282 (32)	0.57	104 (18.8)	73 (11.8)	0.00	279 (22)	207 (17.2)	0.00	208 (17.1)	187 (14.9)	0.13
OAD + Insulin n (%)	233 (22.5)	184 (20.9)	0.39	76 (13.8)	49 (7.9)	0.00	201 (15.9)	140 (11.6)	0.00	142 (11.7)	122 (9.7)	0.11
Statins n (%)	665 (64.3)	529 (60)	0.06	306 (55.4)	348 (56.3)	0.76	774 (61.1)	712 (59.1)	0.32	681 (56)	707 (56.2)	0.90
Diuretics n (%)	249 (24.1)	249 (28.3)	0.04	146 (26.4)	186 (30.1)	0.17	361 (28.5)	370 (30.7)	0.22	299 (24.6)	337 (26.8)	0.21
Beta-blocker n (%)	159 (15.4)	158 (17.9)	0.13	87 (15.8)	121 (19.6)	0.09	195 (15.4)	214 (17.8)	0.11	165 (13.6)	201 (16)	0.09
Calcium channel blocker n (%)	243 (23.5)	195 (22.1)	0.49	118 (21.4)	143 (23.1)	0.47	326 (25.7)	291 (24.2)	0.37	244 (20.1)	249 (19.8)	0.87
ACE n (%)	407 (39.3)	378 (42.9)	0.11	245 (44.4)	281 (45.5)	0.71	559 (44.1)	561 (46.6)	0.22	461 (37.9)	484 (38.5)	0.76
ARB n (%)	288 (27.8)	231 (26.2)	0.43	169 (30.6)	178 (28.8)	0.50	398 (31.4)	347 (28.8)	0.16	307 (25.2)	283 (22.5)	0.11
Antiplatelet n (%)	742 (71.7)	612 (69.5)	0.29	358 (64.9)	401 (64.9)	0.99	891 (70.3)	827 (68.7)	0.38	796 (65.5)	808 (64.3)	0.54

*CAD* Coronary arterie disease, *OAD* Oral antidiabetes drug, *ACE* Angiotensin converting enzyme inhibitor, *ARB* Angiotensin receptor blocker

Among patients with SBP ≥130 mmHg a statistically significant increased use of oral antidiabetic drugs (OAD) and insulin was found in those in the SNCP group. Similar findings were seen in patients with DBP ≥80 mmHg at baseline. Finally, patients with LDL cholesterol ≥100 mg/dl at baseline who were followed in the SNCP group had lived with DM for longer, had retinopathy, and a more frequently used OADs than those in the UNC group.

Overall, the patients in the SNCP group had a higher prevalence of poor personal health habits, older age, a larger number of complications related to T2DM, and had more frequently received treatment for DM (OAD, insulin) and lipid-lowering drugs (statins).

A high percentage of participants − 94.4%- did not achieve all of their ABC goals. No differences were seen between patients aged < 75 years and those aged ≥75 years, although there were differences between genders (males 93.2% vs. females 95.4%, *p* = 0.003). The BP goal (< 130/80 mmHg) was not achieved in 69.2% patients, with statistically significant differences between age groups (68.2% in < 75 years vs. 71.3% in ≥75 years, *p* = 0.045) and genders (66.8% in males vs. 71.4% in females, *p* = 0.001).

A1C ≥7% was recorded in 45.51% of 4210 participants at baseline (*n* = 1916). Of the 881 participants in the UNC group who had A1C ≥7% at baseline, 275 (31.2%) achieved A1C < 7% after 4 years of follow-up vs. 315 of the 1035 participants in the SNCP group with A1C ≥7% at baseline (30.4%). This difference was not statistically significant (*p* = 0.713).

The predictors of achieving A1C < 7% after multivariable analysis are shown in Table 2. SNCP did not show any effect with this goal (OR = 0.97; 95% CI, 0.79–1.19). However, the only variables that was directly and significantly associated was: age (OR = 1.02; 95% CI, 1.01–1.03). The factors that were inversely associated with achievement of A1C < 7% were: duration of DM (OR = 0.98, 95% CI, 0.96–0.99), use of Insulin (OR = 0.27, 95% CI, 0.15–0.49), and use of insulin combined with OAD (OR = 0.31; 95% CI, 0.62–0.98).

**Table 2:** Predictors of A1C < 7%, Among 1916 Patients Who Did Not Achieve A1C Goals at Baseline after Four-year follow-up (Multivariable Logistic Regression)

Variables	aOR	OR 95% CI	*p* value
Nursing Care Plans (SNCP/ UNC)	0.97	0.79–1.19	0.761
Gender (male/female)	1.20	0.96–1.50	0.102
Age (years)	1.02	1.01–1.03	0.001
Duration of diabetes mellitus (years)	0.98	0.96–0.99	0.005
OAD (yes/no)	0.73	0.45–1.17	0.190
Insulin (yes/no)	0.27	0.15–0.49	0.000
OAD + insulin (yes/no)	0.31	0.19–0.53	0.000
BMI < 30 kg/m^2^ (yes/ no)	0.78	0.62–0.98	0.030

Adjusting for diuretics, statins, ACE inhibitors, beta-blockers, calcium antagonists, smoking, arterial hypertension, dyslipidemia, and CAD

With respect to the goal of SBP < 130 mmHg, 274 (13%) participants in the UNC group and 173 participants (8.2%) in the SNCP group were excluded for not having a BP measurement at baseline and at the end of follow-up. Of the 1204 participants in the UNC group who had SBP ≥130 mmHg at baseline, 345 (28.7%) achieved SBP < 130 mmHg at the final visit vs. 372 from 1267 participants (29.4%) in the SNCP group with SBP ≥130 at baseline. This small difference in favor of the SNCP group did not reach statistical significance (*p* = 0.699). The main variables associated with SBP < 130 mmHg after 4 years of follow-up were: BMI < 30 kg/m^2^ (OR = 1.36; 95% CI, 1.12–1.66), and CAD (OR = 1.38; 1.02–1.87). In addition, an inverse correlation was found between use of calcium antagonists or use of insulin combined with OAD and SBP < 130 mmHg (Table 3) .

**Table 3:** Predictor Factors for SBP < 130 mmHg and DBP < 80 mmHg, among T2DM patients did not achieve BP goal at baseline after four-year follow-up (Multivariable Logistic Regression)

Variables	SBP < 130 mmHg (*n* = 2147)			DBP < 80 mmHg (*n* = 1170)		
	aOR	OR 95% CI	*p* value	aOR	OR 95% CI	*p* value
Nursing Care Plans (SNCP/ UNCP)	1.03	0.86–1.23	0.783	1.12	0.88–1.43	0.357
Gender (male/female)	1.07	0.88–1.30	0.492	1.08	0.83–1.40	0.557
Age (years)	1.01	0.99–1.02	0.106	1.05	1.03–1.06	0.000
Duration of Diabetes Mellitus (years)	1.01	0.99–1.02	0.883	1.01	0.99–1.03	0.500
OAD (yes/no)	0.81	0.64–1.03	0.082	1.04	0.76–1.41	0.818
Insulin (yes/no)	0.86	0.56–1.32	0.482	2.13	1.06–4.27	0.033
OAD + Insulin (yes/no)	0.60	0.43–0.85	0.004	0.96	0.59–1.56	0.879
BMI < 30 Kg/m^2^ (yes/ no)	1.36	1.12–1.66	0.002	1.42	1.08–1.87	0.012
Calcium antagonists (yes/no)	0.70	0.56–0.87	0.002	1.01	0.75–1.36	0.947
CAD (yes/no)	1.38	1.02–1.87	0.035	1.43	0.87–2.38	0.162

Adjusting for diuretics, statins, ACE, beta-blocker, tobacco, arterial hypertension, and dyslipidemia

Of the 618 participants in the UNC group who had DBP ≥80 mmHg at baseline, 329 (53.2%) achieved DBP < 80 mmHg at the end of follow-up vs. 322 out of 552 patients (58.3%) in the SNCP group who started with DBP ≥80 mmHg. This increase of five percentage points in favor of the SNCP group did not reach statistical significance (*p* = 0.08).

Table 3 shows that BMI < 30 kg/m^2^ is associated with optimal control of DBP (OR = 1.42; 95% CI, 1.08–1.87). However, CAD showed a positive trend for optimal DBP but was notstatistically significant (OR = 1.43; 95% CI, 0.87–2.38). Use of insulin was shown to be a strong predictor of DBP < 80 mmHg (OR = 2.13; 95% CI, 1.06–4.27).

Finally, of the 1257 participants in the UNC group who had LDL-C ≥ 100 mg/dl at baseline, 508 (40.4%) achieved LDL-C < 100 mg/dl at the end of follow-up vs. 472 (38.8%) out of 1216 patients in the SNCP group who started with LDL ≥100 mg/dl. This disadvantage of SNCP did not reach statistical significance (*p* = 0.417).

The predictor factors for LDL-C < 100 mg/dl after 4 years of follow-up are shown in Table 4. The use of statins (OR = 1.66; 95% CI, 1.36–2.03), treatment of DM with OAD (OR = 1.71; 95% CI, 1.38–2.13), treatment of DM with insulin combined with OAD (OR = 1.91; 95% CI, 1.38–2.64), male sex (OR = 1.49; 95% CI, 1.24–1.78) and having a history of CAD (OR = 1.47; 95% CI, 1.06–2.02) were directly and significantly associated with good control of LDL-C.

**Table 4:** Predictors of LDL-C < 100, Among 2473 Patients Who Did Not Achieve their LDL-C goal at Baseline after Four-year Follow-up (Multivariable Logistic Regression)

Variables	aOR	OR 95% CI	*p* value
Nursing Care Plans (SNCP/ UNCP)	0.90	0.76–1.06	0.217
Gender (male/female)	1.49	1.24–1.78	0.000
Age (years)	1.01	0.99–1.02	0.728
Duration of diabetes mellitus (years)	1.01	0.99–1.02	0.386
OAD (yes/no)	1.71	1.38–2.13	0.000
Insulin (yes/no)	1.51	0.99–2.30	0.053
OAD + Insulin (yes/no)	1.91	1.38–2.64	0.000
BMI < 30 Kg/m^2^ (yes/ no)	0.92	0.77–1.11	0.393
Statins (yes/no)	1.66	1.36–2.03	0.000
Arterial hypertension (yes/no)	1.21	0.98–1.50	0.077
Diuretics (yes/no)	1.18	0.96–1.44	0.111
CAD (yes/no)	1.47	1.06–2.02	0.019

Adjusting for calcium antagonists, ACE inhibitors, beta-blockers, smoking, and dyslipidemia

## Discussion

The present study shows that T2DM patients who were poorly controlled at baseline did not achieve their ABC goals if they were in the SNCP group compared with the UNC group. However, we did observe a trend toward achieving DBP < 80 mmHg in the SNCP group compared with the UNC group.

Early findings from this research project showed that patients in the SNCP group achieved a persistent and significant reduction in DBP, but not in SBP, compared with patients in the UNC group [18]. This improvement in DBP values but not in SBP values reflects the greater difficulty in controlling SBP than DBP, which is highlighted in other studies [24]. In addition, health professionals frequently consider older patients to have good BP control if they reach the DBP goal (< 80 mmHg) even if SBP is above 130 mmHg [25].

In Spain, a similar PHC-based study assessing the outcomes reached over 9 years [26] showed better outcome indicators in chronically ill patients assigned to nurses who implemented care plans than in patients assigned to nurses who did not implement care plans. Specifically, patients in the first group showed higher levels of A1C ≤7% (66.7% vs. 60.3%), BP < 140/90 mmHg (53.3% vs. 50.6%), and total-cholesterol ≤200 mg/dl (39.4% vs. 35.6%; *p* < 0.05) than the second group. A potential explanation for the discrepancy between these findings and ours are the different clinical indicators used to define good control and the inclusion criteria (only patients with poor control in the current study vs. all patients in the study by Pérez Rivas et al.) [26].

At baseline, 94.4% of T2DM patients did not meet all three ABC goals. This figure is similar to that found in other studies such as the National Health and Nutrition Examinatin Survey (NHANES) [27] in 1999–2002 and an Israel cohort study [28]. However, more recently, in NHANES 2007–2010 the percentage of patients who did not meet all of their ABC fell to 81.2% [27].

Non-optimal baseline control of A1C (≥7%) was recorded in 45.1% of participants, consistent with other national studies [29] and international studies [30, 31]. Among people with A1C ≥ 7%, both groups showed improvement in control of A1C from baseline, although the differences were not significant. In both groups a third of patients achieved A1C < 7% after 4 years of follow-up. This improvement is particularly hard to achieve, because the longer a patient has lived with T2DM the more difficult it is to achieve glycemic control [32–34].

The predictive factors for attaining A1C < 7% are concordant with results from previous studies showing that patients who have been treated with insulin (alone or combined with OADs) for longer periods showed poor control of A1C [35]. In contrast with other studies [36], we found that BMI < 30 kg/m^2^ was not a predictior of optimal glycemic control. Baseline control of SBP was non-optimal (≥130 mmHg) in 69.2% of cases; that of DBP was non-optimal in 32% of cases (≥80 mmHg). An controlled BP (≥130/80 mmHg) was recorded in 69.2% of participants. These percentage are higher than those found in NHANES 2007–2010, NHANES 2003–2006, and NHANES 1999–2002. A possible explanation is that the mean age of the patients included in these studies was below 60 years whereas in our study it was 69.2 years. On the other hand, our data are similar to those from the NHANES 1988–1994 study [27] and from the National Diabetes Health Promotion Centers survey in Taiwan [31], where participants were aged over 60 years. This finding is consistent with the known inverse relationship between older age and control of arterial hypertension [37, 38].

Optimal control of SBP at the end of the follow-up was achieved by 29.4% patients in the SNCP group vs. 28.7% in the UNC group. This difference was not statistically significant.

The strongest predictive factor for SBP < 130 mmHg was history of CAD, followed by BMI < 30 kg/m^2^. A recent clinical-epidemiological study of 55,518 primary care patients in Germany [39] found that previous CAD was a significant predictor of adequate BP control (adjusted OR = 1.52; 95% CI, 1.13–1.39). The benefits of weight loss on control of BP, regardless of drug treatment, are well known [40]. The Trial of Hypertension Prevention (TOHP) [41] showed that an average weight loss of 2 kg. was associated with a drop in SBP/ DBP of 3.7/2.7 mmHg. The SNCP group worked on weight reduction, which had no effect on control of SBP possibly because weight loss requires intense interventions (low-calorie diet plus regular physical activity and, in some cases, behavior therapy) as highlighted in the NIC collection [42].

With respect to patients with non-optimal control of DBP at baseline, adequate control was achieved by 58.3% patients in the SNCP group vs. 53.2% in the UNC group. While not statistically significant, this difference seems clinically relevant.

As with SBP, a history of CAD and BMI < 30 kg/m^2^ are shown to be independent predictors of adequate control of DBP. Older age was also positively associated with good control. This finding was previously reported in patients who were of normal weight or overweight [43], although it is not common [44, 45] because older age is usually associated with increased morbidity and poorer control of BP [46].

The percentage of T2DM patients who reached LDL-C < 100 mg/dl was not better in the SNCP group than in the UNC group. Stronger predictors for achieving the LDL-C goal were the administration of statins, treatment with OAD, treatment with insulin combined with OAD, male sex and having a history of CAD. The persistent strength of CAD as a predictor of control of BP and achievement of LDL-C goals could be explained by a self-perception of illness that is more serious and linked to higher medication adherence [47]. It is also possible that patients with previous CAD may have been managed more aggressively [38] than patients without myocardial ischemia.

Long cohort studies typically have high rates of loss to follow-up that potentially affects their validity [48]. Our study had only 15% losses to follow-up, because only patients with at least two HbA1c values during the follow-up period were selected. These patients usually receive better quality care and are more likely to have a second LDL-C than the general population with T2DM.

Our study is subject to a series of limitations. First, the sample was composed of T2DM patients who regularly visited PHC centers and may therefore not be representative of the entire T2DM patient community. However, as we mentioned above, the proportion of T2DM patients in our study who met their ABC goals is similar to that reported elsewhere, and it seems that the potential for selection bias is low. Second, since the quality of evidence from cohort studies is lower than that from clinical trials, our results should be interpreted with caution. Third, the fact that SNCP has been implemented progressively in recent years [26], could reasonably affect our results. Therefore, we carried out an ITT analysis to determine the effect of SNCP; this analysis might have influenced the weaker effect seen in the SNCP group than in the UNC group. However, ITT is the most appropriate study design for this context and is considered standard practice by CONSORT (Consolidated Standards of Reporting Trials). Fourth, we did not control the time the patient remained in each of the study groups. There may have been some crossover that reduced the differences between groups, because there has been significant movement between nursing teams in recent years in Madrid. Furthermore, given the economic recession, nursing teams have become smaller, resulting in nurses having less time with their patients. Fifth, the fact that nursing staff do not receive incentives to improve patient health outcomes could result in less motivation from staff.

Finally, to our knowledge, no studies that have evaluated the effectiveness of SNCP in reaching ABC goals in patients with poorly controlled T2DM at baseline. For this reason, our study is not comparable to other studies with similar efficacy. Further research in this area should be carried out.

## Conclusions

We conclude that there is not enough evidence to favor SNCP over UNC with the aim of helping patients with poorly controlled T2DM at baseline to achieve their ABC goals However, SNCP shows a clear trend to improving the proportion of patients who achieve DBP goals.

## Abbreviations

A1C: Glycosylated hemoglobin
ADA: The American Diabetes Association
BMI: Body mass index
BP: Blood pressure
CAD: Coronary artery disease
CCR: Computerized Clinical Records
DBP: Diastolic blood pressure
DM: Diabetes mellitus
HDL: High-density lipoprotein
LDL: Low-density lipoprotein
NANDA: NANDA-International
NIC: Nursing interventions classification
OAD: Oral antidiabetic drug
PHC: Primary health care
SBP: Systolic blood pressure
SNCP: Standardized languages in Nursing Care Plans
T2DM: Type 2 diabetes mellitus
TOHP: Trial of Hypertension Prevention
UNC: Usual Nursing Care
